# Caspase-2 Maintains Bone Homeostasis by Inducing Apoptosis of Oxidatively-Damaged Osteoclasts

**DOI:** 10.1371/journal.pone.0093696

**Published:** 2014-04-01

**Authors:** Ramaswamy Sharma, Danielle Callaway, Difernando Vanegas, Michelle Bendele, Marisa Lopez-Cruzan, Diane Horn, Teja Guda, Roberto Fajardo, Sherry Abboud-Werner, Brian Herman

**Affiliations:** 1 Department of Cellular and Structural Biology, University of Texas Health Science Center, San Antonio, Texas, United States of America; 2 Department of Pathology, University of Texas Health Science Center, San Antonio, Texas, United States of America; 3 Department of Orthopedics, University of Texas Health Science Center, San Antonio, Texas, United States of America; 4 Department of Biomedical Engineering, University of Texas at San Antonio, San Antonio, Texas, United States of America; Innsbruck Medical University, Austria

## Abstract

Osteoporosis is a silent disease, characterized by a porous bone micro-structure that enhances risk for fractures and associated disabilities. Senile, or age-related osteoporosis (SO), affects both men and women, resulting in increased morbidity and mortality. However, cellular and molecular mechanisms underlying senile osteoporosis are not fully known. Recent studies implicate the accumulation of reactive oxygen species (ROS) and increased oxidative stress as key factors in SO. Herein, we show that loss of caspase-2, a cysteine aspartate protease involved in oxidative stress-induced apoptosis, results in total body and femoral bone loss in aged mice (20% decrease in bone mineral density), and an increase in bone fragility (30% decrease in fracture strength). Importantly, we demonstrate that genetic ablation or selective inhibition of caspase-2 using zVDVAD-fmk results in increased numbers of bone-resorbing osteoclasts and enhanced tartrate-resistant acid phosphatase (TRAP) activity. Conversely, transfection of osteoclast precursors with wild type caspase-2 but not an enzymatic mutant, results in a decrease in TRAP activity. We demonstrate that caspase-2 expression is induced in osteoclasts treated with oxidants such as hydrogen peroxide and that loss of caspase-2 enhances resistance to oxidants, as measured by TRAP activity, and decreases oxidative stress-induced apoptosis of osteoclasts. Moreover, oxidative stress, quantified by assessment of the lipid peroxidation marker, 4-HNE, is increased in *Casp2^-/-^* bone, perhaps due to a decrease in antioxidant enzymes such as SOD2. Taken together, our data point to a critical and novel role for caspase-2 in maintaining bone homeostasis by modulating ROS levels and osteoclast apoptosis during conditions of enhanced oxidative stress that occur during aging.

## Introduction

Bone is a dynamic tissue that undergoes continual remodeling during life [1] through concerted actions of bone-resorbing osteoclasts and bone-forming osteoblasts. A higher bone formation activity will result in a net increase in bone mass whereas a higher bone resorption activity will result in a net loss of bone mass. Post-menopausal, estrogen-deficiency-related bone loss (type I) has been the focal point of osteoporosis research whereas little is known about age-related, senile (type II) osteoporosis. Type I osteoporosis occurs late in life and develops relatively rapidly, following a dip in estrogen levels after menopause and mostly affects cancellous bone. Conversely, age-related osteoporosis begins earlier in life, is independent of sex steroid levels, occurs gradually, and affects cortical and trabecular bone in both men and women [reviewed in 2]. More than 50% of women and 20% of men over the age of 65 are at high risk for senile osteoporosis that is associated with vertebral and hip fractures costing billions of dollars in treatment annually; importantly, osteoporotic fractures also result in increased mortality [3]. Yet, the molecular mechanisms underlying age-related osteoporosis are not fully known.

Recent studies implicate the accumulation of reactive oxygen species (ROS) and increased oxidative stress as key factors in age-related osteoporosis [2], [4], [5]. For example, accumulation of advanced glycation end-products as a result of oxidative stress increases fracture risk [6]. Moreover, decreased bone mineral density (BMD) is associated with higher oxidative stress index values (plasma lipidation) and total plasma oxidant status in osteoporotic patients [7], [8]. In addition, plasma levels and activities of anti-oxidant enzymes such as superoxide dismutase and glutathione peroxidase, negatively correlate with lumbar BMD in humans [9], [10]. Ovariectomy enhances bone resorption due to estrogen insufficiency which correlates to decreased antioxidant levels and increased oxidative stress [11], [12], [13]; treatment with anti-oxidants such as catalase or N-acetyl cysteine prevents such bone loss [12], [14]. Strikingly, bone loss in aged C57BL/6 mice is also associated with increased oxidative stress in bone [14]. These data clearly delineate the deleterious effects of high oxidative stress on bone.

Among the major bone cell types, osteoclasts require low levels of ROS for differentiation and function [15], [16]. Indeed, *in vivo* bone resorption occurs preferentially in sites where ROS and hence, oxidative stress levels are high [11], [12], [15], [16]. However, beyond a certain threshold, it is expected that chronic exposure to elevated oxidative stress will result in cytotoxic effects due to increased oxidative damage of DNA, proteins and lipids that can then lead to apoptosis via caspases.

Caspase-2, the first mammalian apoptotic caspase identified [17], [18], is the most highly conserved caspase across species [19]. It can act as an upstream initiator of mitochondrial permeabilization [20], thereby playing a pivotal role in the initiation of apoptosis via the intrinsic apoptotic pathway. Caspase-2 is critical for oxidative stress-induced apoptosis in several cell types [21], [22], [23] and cells lacking caspase-2 are protected against treatment with mitochondrial complex I inhibitors such as rotenone from apoptosis [22]. Conversely, antioxidants such as NADPH inhibit the activation of caspase-2 [24], suggesting that caspase-2 is regulated by redox state. We have previously shown that old male *Casp2^−/−^* mice (24–26 month old) exhibit significant vertebral bone loss, characterized by increased osteoclast numbers *in vivo,* a two-fold increase in skeletal uptake of a bone scanning agent (Technetium-99 m methylene diphosphonate) that accumulates at active bone remodeling sites, and increased levels of urinary deoxypyridonoline, a bone resorption marker [25]. Therefore, we hypothesized that caspase-2 plays a critical role in apoptosis of aging, oxidatively-damaged osteoclasts.

In this manuscript, we demonstrate elevated caspase-2 expression in osteoclasts following oxidative stress and show that loss of caspase-2 promotes osteoclast survival. Our data point to a novel and critical role for caspase-2 in regulating osteoclast numbers in bone and in preventing bone loss.

## Materials and Methods

### Ethics Statement

All animal procedures were approved by the IACUC (Protocol # 07074z) at the University of Texas Health Science Center at San Antonio in accordance with NIH guidelines.

### Mice

*Casp2^−/−^* mice were generated by Dr. Junying Yuan at Harvard Medical School [26] and backcrossed with C57BL/6J for 10 generations. Only male mice were used in this study. Mice were housed in a pathogen-free, temperature-controlled environment at the AAALAC-accredited animal facility at the University of Texas Health Science Center at San Antonio.

### Bone mineral density (BMD) measurements

Dual-energy X-ray absorptiometry (DXA) was used to measure BMD. Mice were anesthetized with an intraperitoneal injection of a mixture of ketamine hydrochloride (KetaSet III, Fort Dodge Animal Health, Iowa) and xylazine hydrochloride (TranquiVed, Vedco Inc., St. Joseph, MO), laid prone on plastic trays that were then placed on a pre-calibrated Lunar PIXImus densitometer (GE Healthcare, Piscataway, NJ) such that the femur was approximately at an angle of 45° to the image area while the tibia was parallel to the side of the image area. The entire mouse was imaged and scanned. Regions of interest (ROI) corresponding to the diaphysis of the femur and tibia was analyzed.

### Micro-computed tomography (μCT)

Samples were scanned in saline on a desktop μCT system (Sky Scan 1172, Kontich, Belgium) with the following settings: 60 kV, 167 μA beam intensity, 0.5 mm aluminum filter, 0.7° rotation step, 4 frame averaging, 1090 ms integration time, 1024 × 1024 pixel matrix, and a 12 μm isotropic voxel dimension. After scanning, noise was removed from the images by eliminating disconnected objects smaller than 4 pixels in size. Two volumes of interest were selected in the metaphyseal and midshaft regions and automated contouring was used to delineate cortical and trabecular bone regions. In the distal femoral metaphysis, the trabecular bone volume of interest (VOI) was positioned 50 slices proximal to the distal growth plate and extended 150 slices in the proximal direction. The VOI conformed to the endosteal boundary. An appropriate and uniform threshold was applied to all specimens after comparing grayscale and binarized images in both groups. For trabecular bone, a grayscale value of 80 in a set of 8-bit slices was set as the threshold. After thresholding, the BV/TV (%), Tb.Th (mm), Tb.Sp (mm) and Tb.N (mm^−1^) were quantified. Cortical bone structure was analyzed over 50 slices centered at the 55% of length (from proximal to distal) position in the femoral diaphysis. Grey values of 106 and 256 were set as the threshold for cortical bone. Cortical bone analyses included the diaphyseal total area (Tt.Ar, mm^2^), bone area (Ct.Ar, mm^2^), cortical bone area fraction (Ct.Ar/Tt.Ar, %), direct transformation cortical thickness (Ct.Th, mm) and total cortical porosity (Po (tot), %).

### Micro-indentation and three-point bending analyses

The bio-mechanical properties of 27-month old *Casp2^+/+^* and *Casp2^−/−^* fresh frozen bones were assessed by micro-indentation (or reference point indentation) and three-point bending analyses. For micro-indentation, all specimens were placed on a micrometer stage and tested on their posterior surfaces using a BioDent^®^ instrument (Active Life Scientific, Inc., Santa Barbara, CA). System calibration was performed using a poly (methyl methacrylate) block to ensure proper functioning and output of the system. Samples were loaded with a 2 N force, at 2 Hz for 10 cycles. Five sites, each 0.5 mm apart and centered around midshaft, were tested. In some instances, the probe broke through the cortical wall of the femur shaft of *Casp2^−/−^* but not *Casp2^+/+^* bones; these data points were not included in the analyses. Three-point bending analyses of bones were performed on an MTS Insight 5 Electromechanical system (MTS Systems Corporation, Eden Prairie, MN) using TestWorks software (version 4.0). The outer and inner pin distances were 8 mm and 4 mm respectively. The test was performed in displacement control mode at a constant rate of 0.5 mm/sec with data collected at a 200 Hz sampling rate for all measurements. Stress calculations were performed by taking into account the accurate cross sectional areas and moments of inertia of each individual sample determined from μCT.

### Bone histology & immunohistochemistry

Mouse femur and tibia were fixed in 10% neutral-buffered formalin, decalcified in 12% EDTA and embedded in paraffin. Sections (5 μm) were stained with hemotoxylin & eosin to evaluate morphology. For 4-HNE immunohistochemistry, bone sections were de-paraffinized, heated in antigen unmasking solution (Dako, Carpinteria, CA) and permeabilized in phosphate-buffered saline (PBS, pH 7.4) containing 0.2% Triton-X-100. Sections were blocked in 1% bovine serum albumin (BSA) for 1 h, incubated with primary antibody (Alpha Diagnostics, San Antonio, TX) overnight at 4°C, followed by incubation in peroxidase-conjugated antibody (Vectastain Elite Reagent, Vector Labs, Burlingame, CA) and in Sigma Fast 3,3′-diaminobenzidine substrate (Sigma, St. Louis, MO). Sections were counterstained with 0.1% Methyl Green in PBS and examined by light microscopy. For quantification, three sections per mouse (n = 3/group) were immunostained twice and analyzed using Image J. Briefly, channels were separated, the blue channel image was inverted and the threshold adjusted to remove nuclear background. Average staining intensity and stained cell counts were measured in several ROI of the same size.

### Osteoclast cultures and treatments

Osteoclasts were cultured from mouse femurs and tibia or differentiated from RAW 264.7 macrophages. Briefly, bone marrow cells were flushed in α-MEM (Invitrogen, Carlsbad, CA), passed through a 70 μm mesh filter and incubated at 37°C for 1 day in α-MEM/10% FBS with 30 ng/ml CSF-1 (R&D Systems, Minneapolis, MN). Non-adherent bone marrow macrophage (BMM) precursors were transferred to glass coverslips in 24-well plates containing 30 ng/mL RANKL (R&D Systems) and 60 ng/mL CSF-1. Cells were incubated for 6 days, with semi-complete change of MEM/FBS containing RANKL and CSF-1 every 3 days. RAW 264.7 macrophages were cultured in 24-well plates in DMEM supplemented with 10% FBS, 4 mM L-glutamine, 1.5 g/L of sodium bicarbonate and 1 mM sodium pyruvate (Invitrogen). The medium was supplemented with 35 ng/mL RANKL to promote osteoclast differentiation. Medium was changed every 3 days. Osteoclasts were identified as TRAP-positive, multinucleated cells. Cell viability was quantified with CellTiter 96 AQueous One Solution Cell Proliferation Assay (G3582, Promega, Madison, WI). In experiments involving transfection of caspase-2, RAW264.7 cells were transferred to 12-well tissue culture plates at a cell density of 2.5×10^5^ cells/well and incubated overnight at 37°C. Wild type or a catalytically inactive (C303S) caspase-2 construct [18] were transfected using Lipofectamine 2000 (Invitrogen) according to the manufacturer's protocols. Osteoclast sensitivity to oxidants was measured after treatment with the general oxidant, H_2_O_2_, or an inducer of mitochondrial radical formation, rotenone (Complex I inhibitor), at the indicated doses. To inhibit caspase-2 activity, osteoclast cultures at day 6 were cultured in the presence of 100 μM zVDVAD-fmk (R&D Systems) or vehicle (0.5% DMSO) for 4 days, stained for TRAP or TUNEL and counted. To determine if zVDVAD-fmk pre-treatment prevented apoptosis, osteoclast cultures were cultured in the presence of 100 μM zVDVAD-fmk (R&D Systems) or vehicle (0.5% DMSO) for 24 h, fixed, TUNEL-stained and counted.

### Tartrate resistant acid phosphatase (TRAP) staining and activity

Osteoclasts on glass coverslips were fixed with 4% paraformaldehyde, and stained for TRAP activity (Sigma) according to the manufacturer's manual. Images were captured using a Nikon Diaphot 300 inverted microscope. TRAP activity was quantified by a colorimetric method. Cells grown in 48-well plates were washed with Hanks Buffer and incubated at 37°C with a pre-warmed, 200 μL mixture containing 0.1% SDS, 2 mg p-nitrophenol phosphate, acetate and tartrate solution (Sigma) for 30 min. The reaction was stopped by adding 40 μL of 0.5 M NaOH and absorbance was read at 405 nm in a microplate reader (BMG Labtech, Cary, NC).

### Immunocytochemistry

Osteoclasts on glass coverslips were treated with H_2_O_2_ or rotenone for the indicated time periods, fixed with 4% paraformaldehyde and blocked with PBS, pH 7.4, containing 1% BSA and 0.3% Triton-X-100 for 1 h at room temperature. After incubation with primary antibodies overnight at 4°C in PBS containing 0.1% BSA and 0.03% Triton-X-100 (rat anti-caspase-2, EMD-Millipore, Billerica, MA), cells were washed and incubated with Alexa-488-conjugated secondary antibodies (Invitrogen) at room temperature for 2 h. Hoechst 33342 (Invitrogen) was used to label nuclei. Coverslips were transferred to slides containing antifade mounting medium (VectaShield, Vector Laboratories, Burlingame, CA), sealed and visualized on an Olympus confocal microscope (Olympus, Japan).

### Immunoblotting

For SOD2 quantification, protein from primary osteoclast cultures at day 4 was extracted in RIPA lysis buffer (sc24948, Santa Cruz Biotechnology, Inc., Santa Cruz, CA), fortified with protease inhibitors (Roche Diagnostics). For caspase-2 expression, RAW 264.7 cells were differentiated into osteoclasts and treated with 500 μM H_2_O_2_ for 2 h or 4 h, followed by protein extraction. Protein concentration was determined using the BCA assay kit (Pierce, Rockford, IL). Proteins (30 μg) were loaded onto 4–20% polyacrylamide gels (Invitrogen), transferred to PVDF membranes, blocked with 5% non-fat dry milk (NFDM) for 1 h and probed overnight at 4°C with anti-SOD2 antibody (sc-30080, Santa Cruz Biotechnology, Santa Cruz, CA) or anti-caspase-2 antibody (MAB3507, Millipore, Billerica, MA) in 1% NFDM. The membranes were then washed and incubated with appropriate HRP-conjugated secondary antibodies (Santa Cruz Biotechnology) for 1 h and developed using an enhanced chemiluminescence system (Pierce, Rockford, IL). Blots were stripped and re-probed for GAPDH (A9521, Sigma) or actin (sc-616, Santa Cruz Biotechnology) as loading controls.

### TUNEL staining

Osteoclasts on glass coverslips were fixed with 4% paraformaldehyde and stained for TUNEL (Roche Diagnostics, Indianapolis, IN) according to the manufacturer's protocols for 30 min at 37°C. After incubation, coverslips were washed and stained with Hoechst 33258 (Invitrogen) for 30 min. Coverslips were visualized on a Nikon Diaphot 300 microscope. TUNEL-positive, condensed nuclei were identified as apoptotic. TUNEL analysis for apoptosis was accomplished by counting a total of 100 osteoclasts per coverslip. Data shown is from an average of 4 independent experiments with H_2_O_2_ treatment and 3 independent experiments with rotenone.

### Osteoclast activity assays

Osteoclasts from wild type and *Casp2^−/−^* mice (n = 3) were differentiated in triplicate on calcium phosphate-coated 48-well plates (Cosmo Bio USA, Inc., Carlsbad, CA). On day 12, media was replaced with 6% sodium hypochlorite for 5 min, wells were washed, dried and imaged. Pits in all wells (3 replicates per mouse) were counted and analyzed for resorption area per pit and intensity of resorption area/pit using Metamorph version 6.3 r7 (Molecular Devices, LLC, Sunnyvale, CA).

### Statistics

Student's *t*-tests were performed using GraphPad Prism version 5.00 (GraphPad Software, San Diego, CA) to determine significance. A *p*-value < 0.05 was considered significant. Data in graphs are reported with standard errors.

## Results

### Casp2−/− mice exhibit age-related bone loss

**Figure 1:**
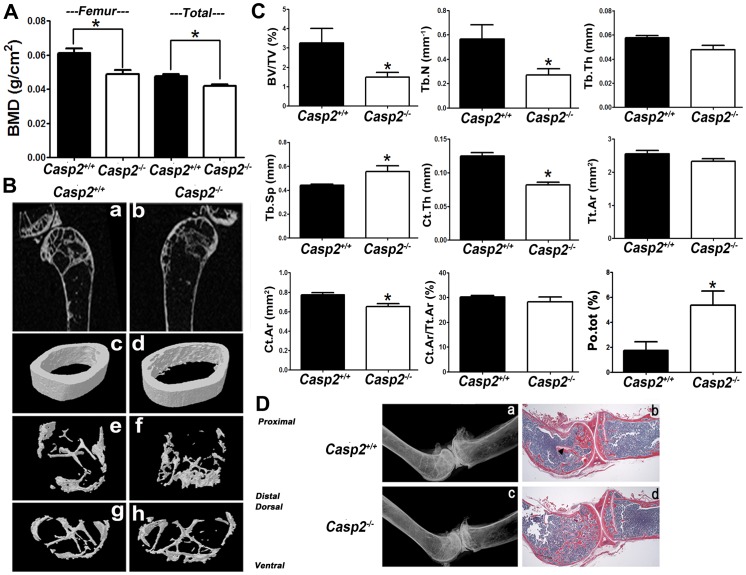
Ablation of caspase-2 results in bone loss. (A) Dual X-ray energy absorptiometry (DXA) analysis indicated a significant decrease in total (n = 10; *, *p* = 0.0017) and femoral (n = 10; *, *p* = 0.0016) bone mineral density (BMD) in 27-month old male *Casp2^−/−^* mice as compared to *Casp2^+/+^* mice. Data represent mean ± SEM. (B) 2D (panels a & b) and 3D (panels c-h) μCT images of 27-month old male *Casp2^+/+^* and *Casp2^−/^* mice showing midshaft femur (panels c & d), lateral view of distal femur (panels e & f) and axial view of distal femur (panels g & h). A higher endocortical porosity and a wider (medio-lateral) metaphyseal compartment are observed (n = 8). (C) Parameters evaluated using μCT (n = 8;*, *p*<0.05); data are represented as mean ± SEM. (D) X-ray (panels a & c) and H&E staining (panels b & d) indicated thinner inter-lacing trabecular bone, and increased cortical bone porosity in *Casp2^−/−^* mice. Arrow indicates growth plate.

DXA analyses of 27-month old mice (Fig. 1A) indicated a significantly lower total body (11.8% decrease) and femoral (20% decrease) bone mineral density (BMD) as compared to wild-type mice (*p* = 0.0017 and *p* = 0.0016 respectively). 2D μCT analysis (Fig. 1B, panels a,b) and reconstructed 3D-images of *Casp2^−/−^* femur (Fig. 1B, panels c,d) showed higher cortical porosity and a wider (medio-lateral) metaphyseal compartment (Fig. 1B, panels e-h) in 27-month *Casp2^−/−^* femurs as compared to *Casp2^+/+^* femur. Several parameters, as described before [27], were analyzed by μCT. Trabecular bone showed significantly decreased bone volume to total volume ratio (BV/TV; p = 0.0251), and decreased trabecular number (Tb.N; p = 0.0288). Trabecular thickness (Tb.Th) trended lower in *Casp2^−/−^* femurs but was not significantly different (p = 0.0608). However, trabecular separation (Tb.Sp) was significantly increased (p = 0.0024). μCT of the midshaft region indicated that total area (Tt.Ar) was unchanged (p = 0.0923) but cortical area (Ct.Ar) decreased significantly (p = 0.0128); the cortical to total area ratio (Ct.Ar/Tt.Ar trended lower (p = 0.0511) in *Casp2^−/−^* femurs. Cortical thickness (Ct.Th) was significantly decreased (p<0.0001) and bone porosity (Po; tot; %) was significantly increased (p<0.05) in the *Casp2^−/−^* femurs.

### Loss of caspase-2 alters biomechanical properties of bone

**Figure 2:**
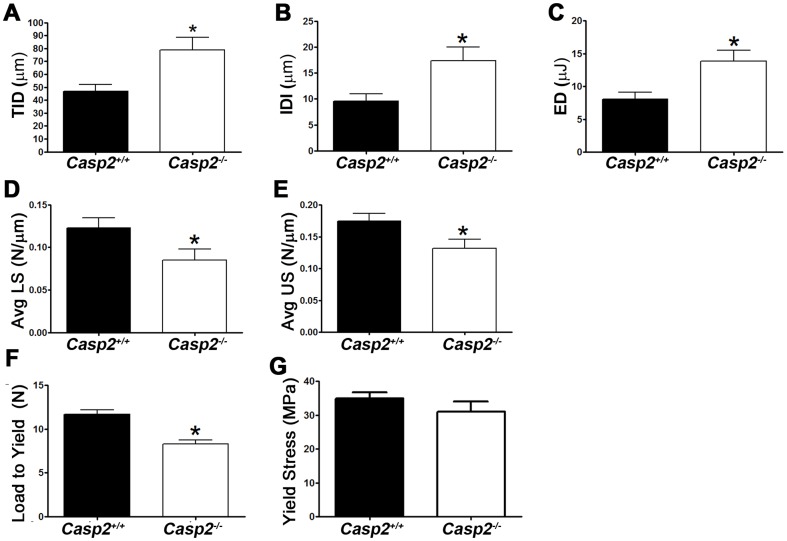
Loss of caspase-2 results in altered biomechanical properties of bone. Parameters measured using micro-indentation analyses (n = 3) include (A) Total indentation distance (TID) (B) Indentation distance increase (IDI), (C) Energy dissipated (ED), (D) Average Loading Slope (Avg LS) and (E) Average Unloading Slope (Avg US). Three-point bending tests (n = 3) were used to measure (F) Load to Yield and (G) Yield Stress. Data are represented as mean ± SEM. *, *p*<0.05.

Because loss of caspase-2 resulted in decreased bone mass, we analyzed the biomechanical properties of *Casp2^−/−^* bone using micro-indentation and three-point bending analyses. We observed a significant increase in all parameters tested (Fig. 2). These included: total indentation distance (TID; *p* = 0.021), indentation distance increase (IDI, *p* = 0.0225), energy dissipated (ED; *p* = 0.015), average unloading slope (Avg US; *p* = 0.0424) and average loading slope (Avg LS; *p* = 0.0331). Three-point bending mechanical tests also showed a significant decrease in Load to Yield (by 30%; *p* = 0.0091); Yield Stress showed a decreasing trend that was not significant. Together, these data suggest decreased bone mass and increased bone fragility in the absence of caspase-2.

### *Caspase-2 regulates osteoclast numbers by promoting apoptosis*

**Figure 3:**
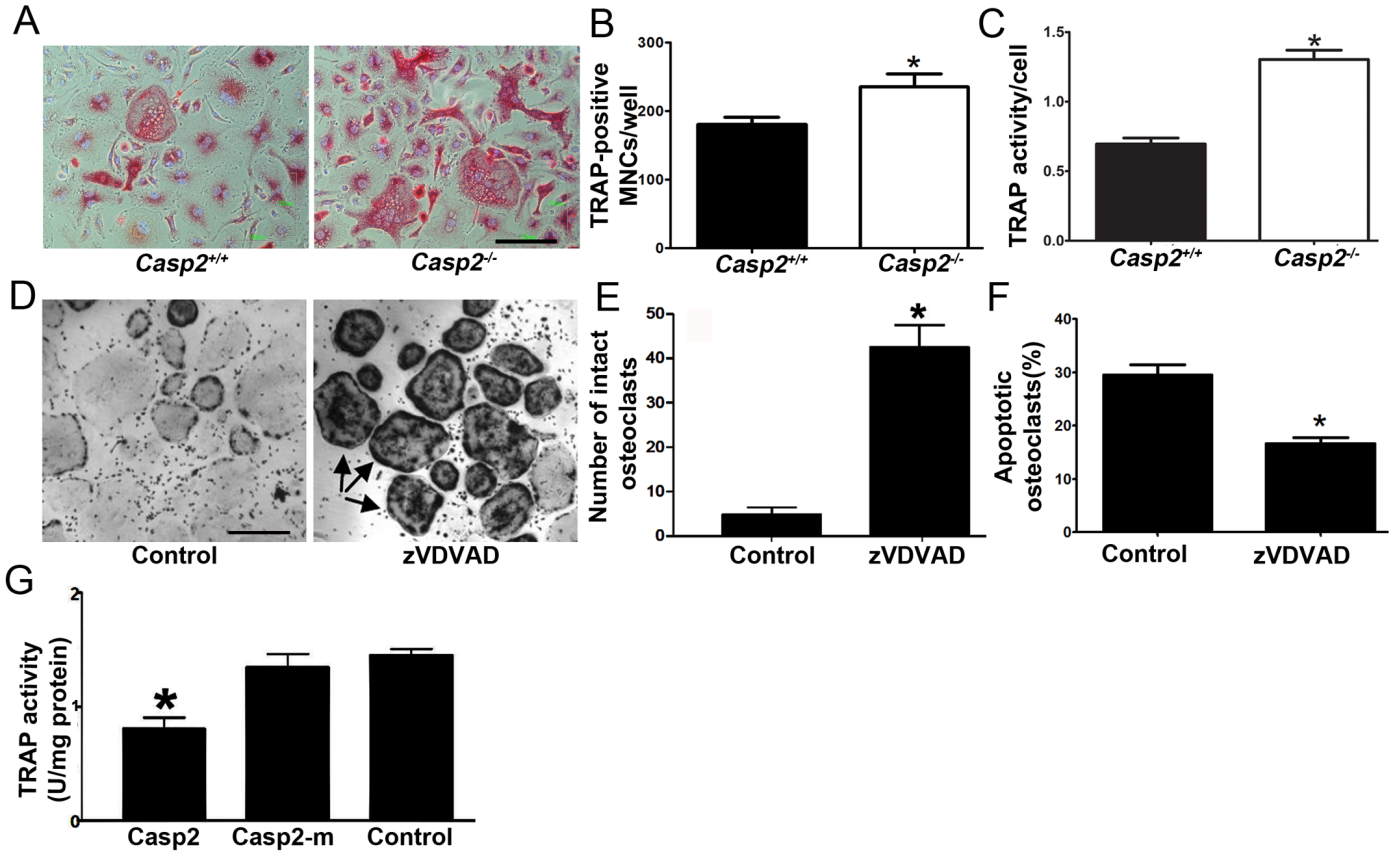
Caspase-2 regulates osteoclast numbers via apoptosis. (A) Primary cultures of *in vitro* differentiated *Casp2^+/+^* and *Casp2^−/−^* osteoclasts grown in 96-well plates were TRAP-stained. (B) TRAP-positive, multinucleated osteoclasts were counted (*, *p*<0.05). (C) TRAP activity of in vitro cultured primary osteoclasts from *Casp2^+/+^* and *Casp2^−/−^* mice was measured and normalized to cell viability as determined by MTT assay (*, *p*<0.0005). (D) Osteoclasts were cultured in the presence of a caspase-2-selective inhibitor, zVDVAD and, (E) Intact TRAP-positive osteoclasts were counted (*, *p*<0.0001). (F) Osteoclasts cultured with or without zVDVAD were stained with TUNEL and counted (*, *p*<0.001). (G) Overexpression of wild type caspase-2 but not its enzymatic mutant (Casp2-m) in the osteoclast precursor cell line, RAW264.7, significantly reduced TRAP activity (*, *p*< 0.005). Scale bar, 100 μm.

The *in vivo* increase in osteoclast numbers, as demonstrated previously [25], was recapitulated *in vitro* by differentiating hematopoietic progenitor cells from *Casp2^+/+^* and *Casp2^−/−^* mice (Fig. 3A, B). Enzymatic activity of tartrate-resistant acid phosphatase (TRAP; a selective marker for osteoclasts) was also up-regulated in *Casp2^−/−^* osteoclast cultures (Fig. 3C). We have previously shown that caspase-2 plays an important role in apoptosis of oxidatively-damaged cells [22]. Because caspase-2 is a pro-apoptotic protein, we hypothesized that caspase-2 mediates osteoclast death. A consequence of caspase-2 deficiency would be the reduced death/or prolonged survival of osteoclasts, that may result in increased bone resorption. To determine if caspase-2 regulates osteoclast numbers by apoptosis, we cultured day 6 osteoclasts in the presence of a selective caspase-2 inhibitor, zVDVAD-fmk, or vehicle only (DMSO) for 4 days. A 7.9-fold increase in the number of intact, viable, TRAP-positive osteoclasts was observed (Fig. 3D, E). To confirm that zVDVAD-fmk prevented apoptosis, osteoclasts were pre-treated with zVDVAD-fmk for 24 h, stained for TUNEL and counted (Fig. 3F). We observed a significant decrease in apoptosis in *Casp2^−/−^* osteoclasts (p = 0.0002). Therefore, caspase-2 may regulate osteoclast apoptosis and loss of caspase-2 can result in increased osteoclast survival. To further confirm a role for caspase-2 in modulating osteoclast numbers, we transfected the osteoclast precursor cell line, RAW 264.7, with catalytically active or mutant caspase-2, followed by differentiation into osteoclasts. As shown in Fig. 3G, overexpression of wild type but not mutated caspase-2 significantly reduced TRAP activity. Taken together, these results indicate that caspase-2 plays an important role in regulating osteoclast numbers by modulating apoptosis.

### Oxidative stress-inducing agents elevate expression of caspase-2 in osteoclasts

**Figure 4:**
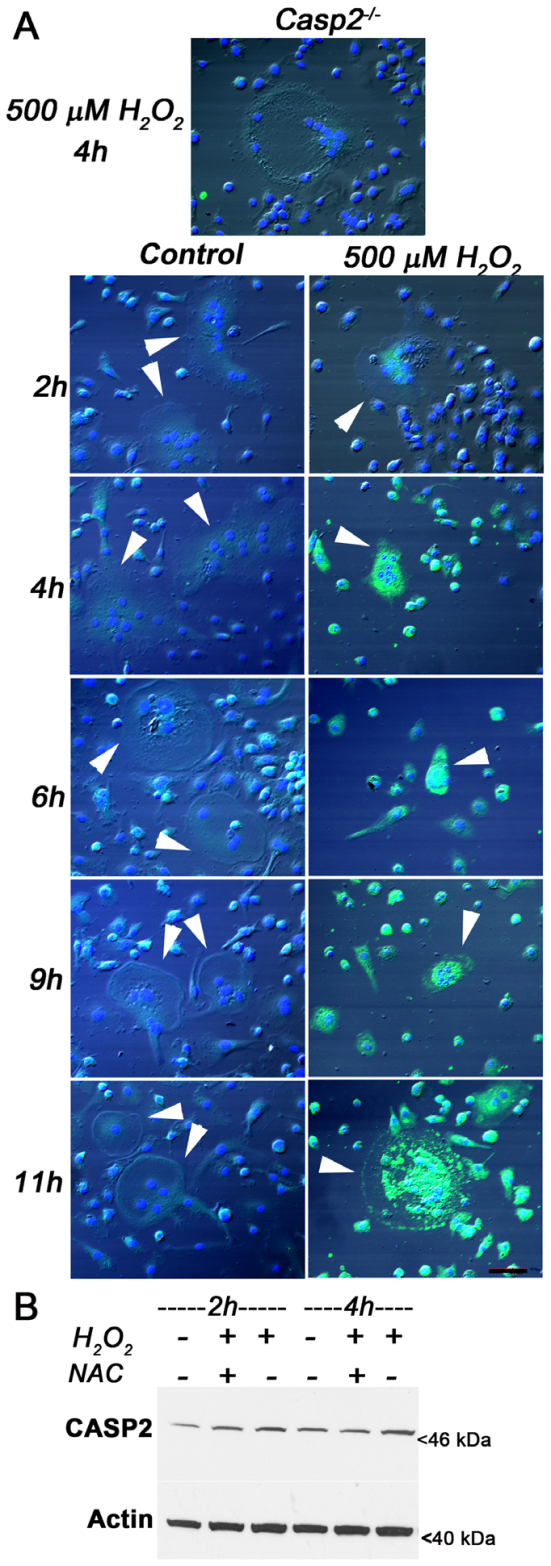
Caspase-2 is induced in osteoclasts on treatment with oxidative stress-inducing agents. (A) Primary cultures of *Casp2^+/+^* osteoclasts treated with 500 μM H_2_O_2_ for the indicated time points (n = 3), were fixed and probed for caspase-2 (green); nuclei were stained blue. *Casp2^−/−^* osteoclasts treated with 500 μM H_2_O_2_ for 4 h served as a negative control. Scale bar, 50 μm. (B) RAW 264.7 macrophages were differentiated into osteoclasts, treated with 500 μM H_2_O_2_ for the indicated time periods, lysed and probed for caspase-2 (n = 3). Stripped blot was probed for total actin as loading control.

Caspase-2 is regulated by redox-regulated factors including NADPH [24], a co-factor of several anti-oxidant enzymes such as glutathione reductase, and calmodulin dependent kinase II [28], [29]. Therefore, to determine if caspase-2 is activated by oxidative stress in osteoclasts, we treated osteoclasts with hydrogen peroxide. Whereas osteoclasts normally expressed very low levels of caspase-2, exposure to hydrogen peroxide triggered the induction of caspase-2 (Fig.4A). Counts indicated that within 2 h of exposure, 47% of osteoclasts expressed high levels of caspase-2 that increased to 82% by 4 h, suggesting that caspase-2 induction preceded cell death. Using immunoblots, we confirmed increased caspase-2 expression after treatment of differentiated osteoclasts with H_2_O_2_. Pre-treatment with 15 mM N-acetyl cysteine for 1 h prior to H_2_O_2_ exposure prevented the observed induction of caspase-2 expression. This suggests that elevation of caspase-2 expression was mediated by oxidative stress.

### Loss of caspase-2 protects osteoclasts from oxidative stress-induced apoptosis

**Figure 5:**
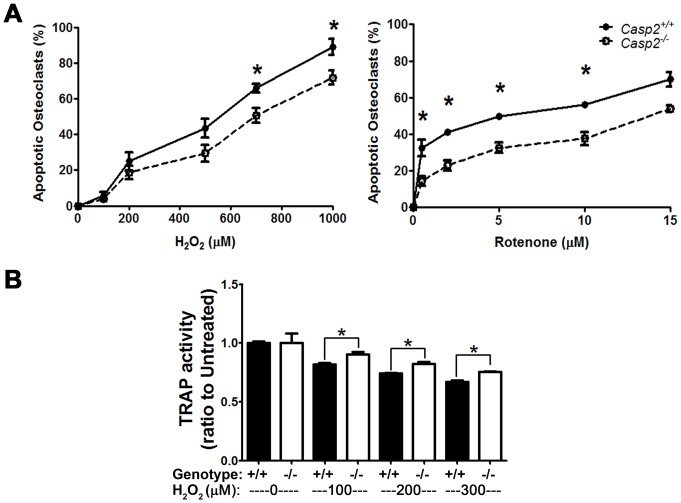
*Casp2^−/−^* osteoclasts are more resistant to oxidative stress. (A) Primary osteoclast cultures of *Casp2^+/+^* and *Casp2^−/−^* mice were exposed to H_2_O_2_ (n = 4) or rotenone (n = 3) for 6 h followed by TUNEL staining. TUNEL-positive osteoclasts with condensed nuclei were considered apoptotic and are presented as a percent of total osteoclasts. *, *p*<0.0001. (B) Primary osteoclast cultures were exposed to the indicated concentrations of H_2_O_2_ for 5 h followed by TRAP activity assay. Results shown are an average of 4 assays (*, *p*<0.001).

Since our data indicated a role for caspase-2 in osteoclast apoptosis, we determined if loss of caspase-2 protects osteoclasts from apoptosis. We treated *Casp2^+/+^* and *Casp2^−/−^* osteoclasts with increasing doses of oxidants (H_2_O_2_ or rotenone) for 6 h and counted apoptotic osteoclasts that were labeled using TUNEL. As expected, a dose-dependent increase in osteoclast apoptosis was observed; strikingly, lower numbers of *Casp2^−/−^* osteoclasts were apoptotic as compared to *Casp2^+/+^* osteoclasts (Fig. 5A; *p*<0.0001 for both treatments). As a measure of osteoclast homeostasis, we quantified TRAP activity after treatment with lower doses of H_2_O_2_ for 5 h (Fig 5B). *Casp2^−/−^* osteoclasts were more resistant to H_2_O_2_ treatment at all doses tested (*p*<0.001). Therefore, caspase-2 plays an important role in osteoclast homeostasis and survival.

### Loss of caspase-2 enhances oxidative stress in bone

**Figure 6:**
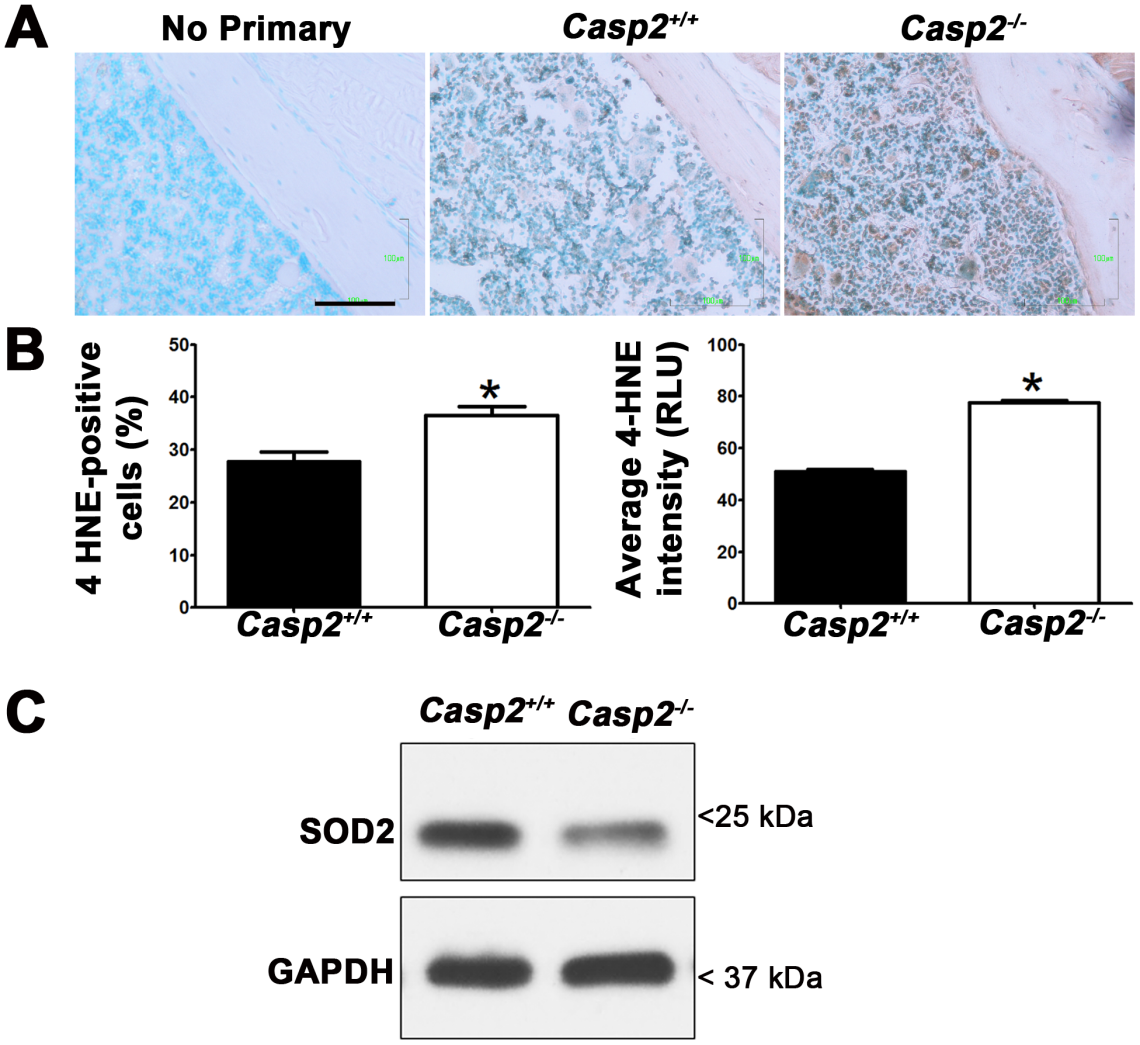
Loss of caspase-2 increases oxidative stress *in vivo*. (A) Bone sections from 27-month old mice (n = 3) were immunostained for 4-HNE; *Casp2^−/−^* section was used as a no-primary control to determine antibody specificity. Data shown is representative of two experiments. Scale bar, 100 μm. (B) Percentage of cells stained (*, *p* = 0.0007) and relative intensities of staining were measured using Image J and plotted (*, *p*<0.0001). (C) Representative western blots of osteoclast lysates probed for the anti-oxidant enzyme, SOD2; GAPDH was used as a loading control.

While caspase-2 is regulated by redox state, caspase-2 may also regulate antioxidants such as sestrins, catalase and superoxide dismutase [30]. Indeed, we have previously shown that loss of caspase-2 in liver results in increased oxidative stress [25]. To determine if loss of caspase-2 elevates overall oxidative stress within the bone microenvironment, we performed immunostaining for 4-HNE (a marker of lipid peroxidation) on *Casp2^+/+^* and *Casp2^−/−^* bone sections (Fig. 6A). We counted the number of cells stained (Fig 6B, left panel) and normalized it to total numbers of cells; more cells in *Casp2^−/−^* bone were immunoreactive for 4-HNE (*p* = 0.0007). We also quantified the intensity of 4-HNE staining using Image J (Fig. 6B, right panel). Staining intensity was significantly higher in bone sections from *Casp2^−/−^* mice (*p*<0.0001). To determine if caspase-2 regulated ROS levels by modulating antioxidants, we quantified SOD2 expression in day 4 osteoclast cultures by western blots. SOD2 expression decreased by 1.5-fold in untreated *Casp2^−/−^* osteoclasts as compared to WT osteoclasts (Fig. 6C).

### Casp2^−/−^ osteoclasts exhibit increased activity

**Figure 7:**
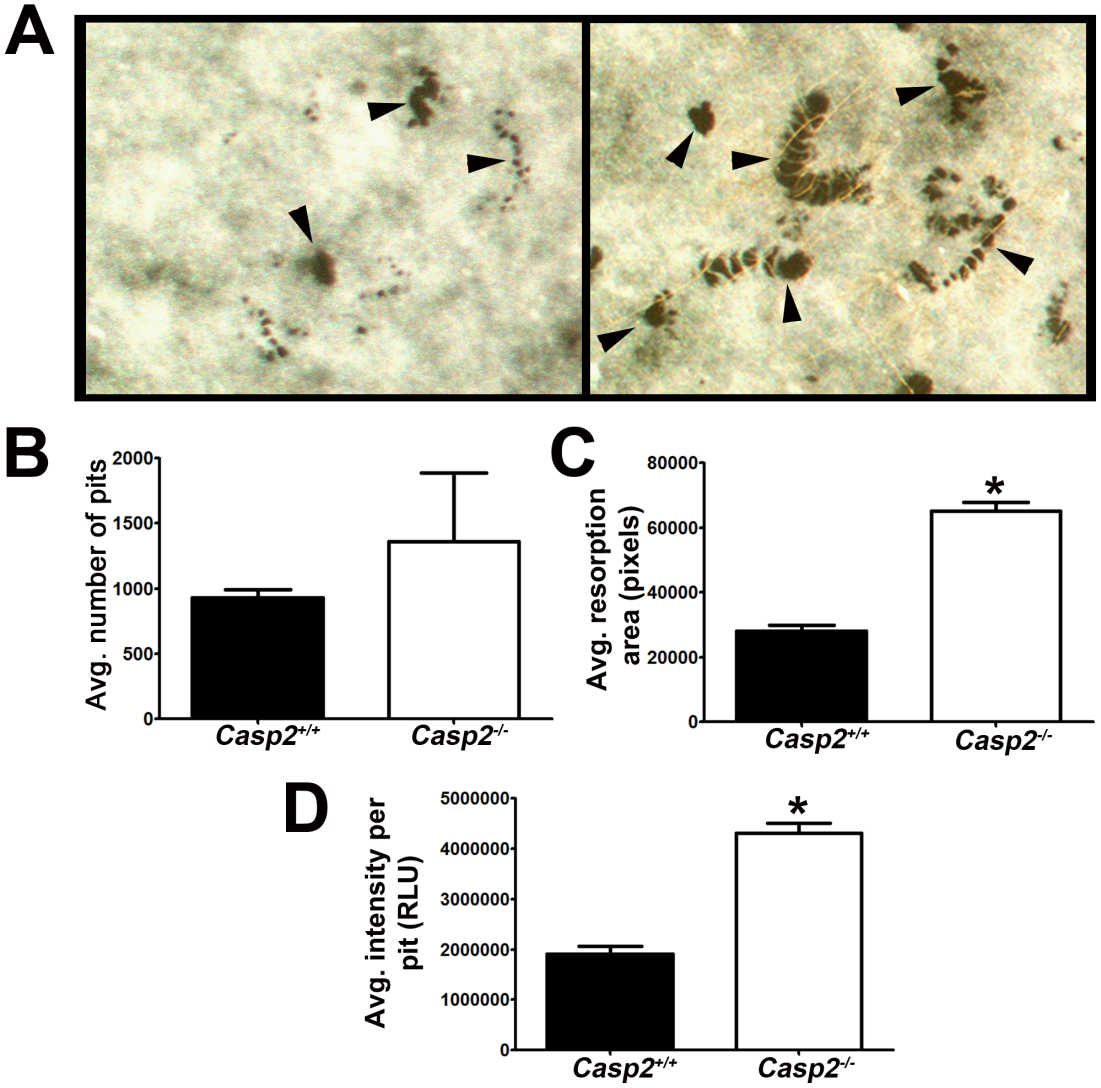
*Casp2^−/−^* osteoclasts exhibit increased resorption. (A) Representative images of resorption pits (arrows) from *Casp2^+/+^* and *Casp2^−/−^* mice. Three wells per mouse and three mice per genotype were analyzed for (B) number of resorption pits, (C) area of resorption pit (*, *p*<0.005) and (D) intensity of resorption pit (*, *p*<0.005). RLU =  Relative Light Units.

We next measured if *Casp2^−/−^* osteoclasts were functionally more active as compared to wild type osteoclasts. Bone marrow macrophages from wild type and *Casp2^−/−^* mice (n = 3; 3 replicates per mouse) were differentiated on calcium phosphate coated 48-well plates to form resorption pits (Fig. 7A). *Casp2^−/−^* osteoclasts exhibited a trend towards increased pit formation (Fig. 7B), and significant increase in resorption surface area (Fig. 7C) and resorption intensity (Fig. 7D).

## Discussion

Since osteoclasts are short-lived cells (2 to 4 weeks), any alteration that prolongs osteoclast viability may result in increased bone resorption, thereby having a pronounced effect on bone homeostasis. Indeed, bisphosphonate-based anti-osteoporosis therapies target osteoclast life-span by promoting their apoptosis [31].

Osteoclast apoptosis may be stimulated by extrinsic signals that activate death receptors on the osteoclast surface, such as the Fas ligand [32], resulting in activation of initiator caspases 8/10, followed by the executioner caspase, caspase-3. Alternatively, intrinsic signals that disrupt mitochondrial membrane integrity will result in the release of cytochrome c, formation of the apoptosome and activation of caspases 9 and 3 [33]. Since osteoclasts are rich in mitochondria that are the major source of ROS, the intrinsic apoptotic pathway is found to be mostly involved in ROS-induced osteoclast apoptosis [33]. Indeed, the role of caspase-3 in osteoclast differentiation and apoptosis is well-established [34], [35]. However, little is known about the function of caspase-2 in osteoclasts. Our *in vitro* data indicate that caspase-2 expression is induced by oxidative stress in osteoclasts (Fig. 4). We find that caspase-2 is expressed in 82% osteoclasts by 4 h whereas 40% osteoclasts are TUNEL-positive at 6 h (Fig. 5), indicating that caspase-2 expression precedes osteoclast apoptosis. Corroborating our data is our finding that loss of caspase-2 increases osteoclast numbers (Fig. 3A) by prolonging survival (Fig. 5). Importantly, we demonstrate that *in vivo* loss of caspase-2 resulted in bone loss (Fig. 1) and consequently bone fragility (Fig. 2) in aged mice. Furthermore, we find that loss of caspase-2 further increases oxidative stress *in vivo* in bone, perhaps due to a reduction in antioxidant expression, such as SOD2 (Fig. 6). The latter is interesting because both SOD1 [36], [37] and SOD2 [38] have been associated with bone loss and, similar to caspase-2, SOD1 has been implicated in mitochondria-dependent pathway of apoptosis [39].

Several laboratories have demonstrated an important role for caspase-2 in oxidative stress-induced apoptosis [21], [23]. We have previously shown that loss of caspase-2 protects fibroblasts and neurons from mitochondrial oxidative stress-induced apoptosis [22]. Moreover, caspase-2 mRNA and enzymatic activity (but not caspases 3 and 6) are rapidly elevated following increased oxidative stress due to estrogen withdrawal [40]. Strikingly, caspase-2 is regulated by redox-regulated factors including NADPH [24], a co-factor of several anti-oxidant enzymes such as glutathione reductase, and calmodulin dependent kinase II (CaMK II) [28], [29]. While these data suggest ROS-mediated activation of caspase-2, caspase-2 may also regulate antioxidants such as sestrins, catalase and superoxide dismutase through the modulation of FoxO transcription factors, thereby negatively modulating ROS [30]. Hence, loss of caspase-2 increases endogenous ROS production, as we have observed in hepatocytes [41] and shown here. Interestingly, FoxO3 is indispensable for skeletal homeostasis [42] and caspase-2 regulates FoxO3a [30], thereby providing strong mechanistic links between caspase-2, oxidative stress and skeletal health.

We posit that caspase-2 plays dual roles in osteoclasts: (i) upregulating anti-oxidants during lower levels of ROS exposure, thereby promoting survival or differentiation and (ii) inducing apoptosis of oxidatively damaged osteoclasts. During aging, a gradual decrease in antioxidant levels will result in increased oxidative stress in bone. Consequently, oxidatively-damaged osteoclasts undergo caspase-2-mediated apoptosis and are cleared. However, in the absence of caspase-2, expression of antioxidants such as SOD2 dip further [30], causing increased oxidative damage to osteoclasts; yet, lack of caspase-2 delays apoptosis of damaged osteoclasts. Interestingly, osteoclasts nearing apoptosis are highly resorptive [43], aggravating bone loss. Our data (Fig. 7) support this observation. Inefficient clearance of oxidatively-damaged osteoclasts may further contribute to oxidative damage in bone, cumulatively providing a positive feedback loop that result in enhanced bone resorption and osteopenia with aging. Of note, we find active caspase-2 within the mitochondrial sub-compartment (unpublished data), suggesting that it could play a critical role under conditions of oxidative stress.

At the molecular level, ROS can damage enzymes responsible for production of NADPH and CaM [28], [29]. In addition, ROS neutralization consumes NADPH. These changes can potentially impair the function of CaMK II, an inhibitor of caspase-2 [24]. It is, therefore, possible that partial loss of CaMK II function reduces the threshold of oxidative stress required for the activation of caspase-2, resulting in osteoclast apoptosis. Interestingly, heterozygous CaMKα II mice have fewer osteoclasts than wild type mice [44], lending support to our hypothesis. In conclusion, our research points towards a novel role for caspase-2 in the maintenance of osteoclasts and thereby, bone homeostasis, during aging.
